# Supplementary material for: Improving the Hydrolysis Rate of the Renewable Poly(hexamethylene sebacate) Through Copolymerization of a Bis(pyrrolidone)-Based Dicarboxylic Acid
Source: Polymers (Basel). 2019 Oct 11;11(10):1654. doi: 10.3390/polym11101654 (PMC6835251; doi:10.3390/polym11101654)
Supplement: Supplementary file 1 [file polymers-11-01654-s001.pdf]

Supporting information for manuscript;

Improving the Hydrolysis Rate of the Renewable Poly(hexamethylene sebacate) Through Copolymerization of a Bis(pyrrolidone)-Based Dicarboxylic Acid.

Authors:

G.J. Noordzij,[a,b] M. Roy,[a], N. Bos[a], V. Reinartz,[a] C.H.R.M. Wilsens,[a]*

[a]*Aachen-Maastricht Institute of Biobased Materials (AMIBM), Faculty of Science and Engineering, Maastricht University, Brightlands Chemelot Campus, 6167 RD Geleen, The Netherlands.*

[b]Chemelot InSciTe, Urmonderbaan 20F, NL-6167 RD Geleen, The Netherlands.
* Corresponding author: karel.wilsens@maastrichtuniversity.nl

5 pages, 5 figures

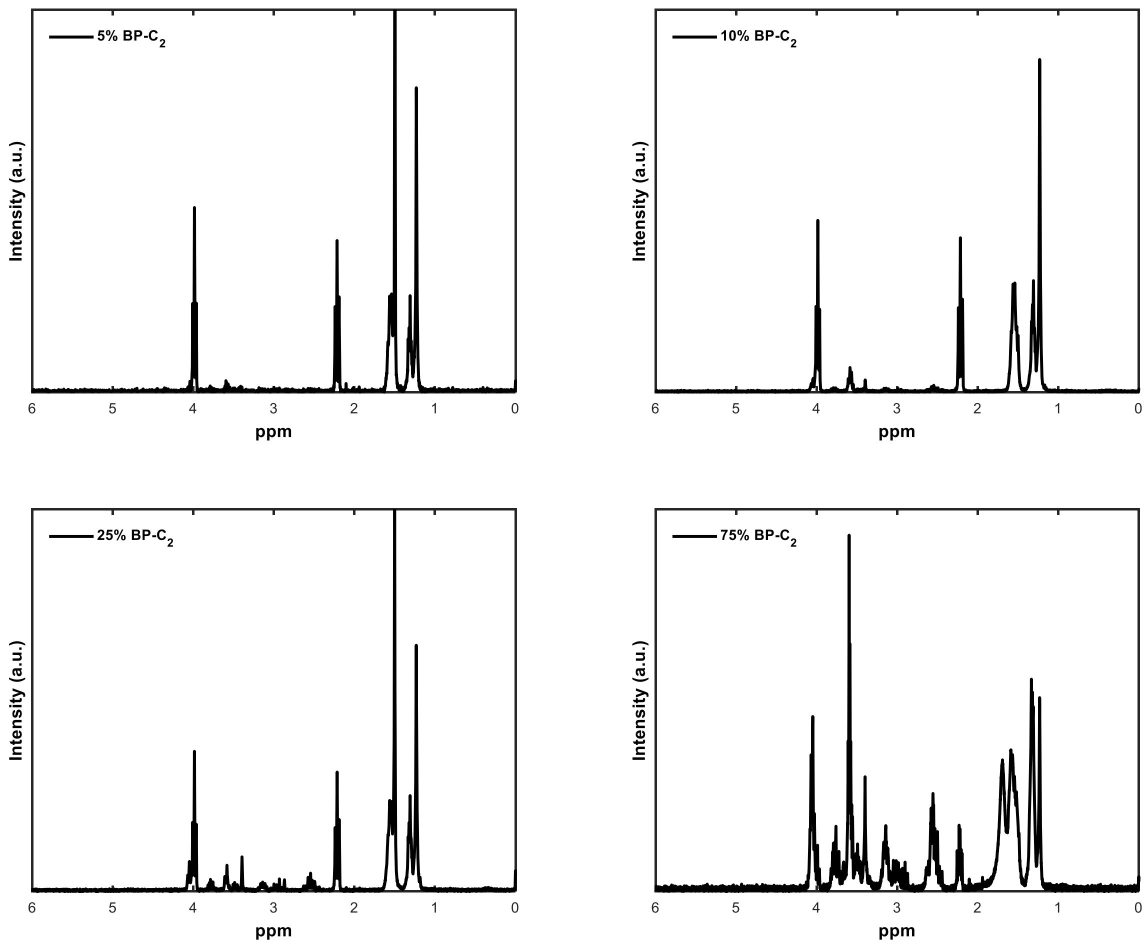

Figure S1. Overview of NMR spectra of the polymers **5% BP-C_2**, **10% BP-C_2**, **25% BP-C_2**, and **75% BP-C_2**.

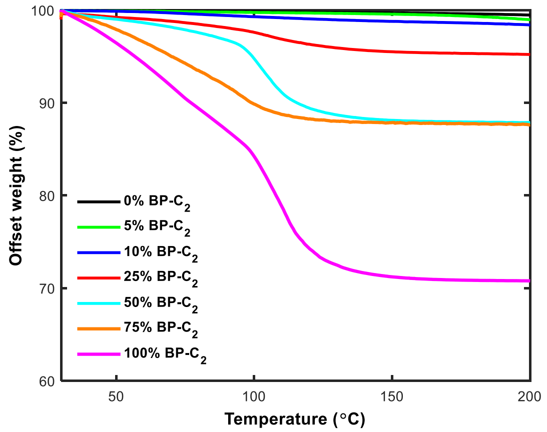

Figure S2. Overview of TGA results of the polymers after immersion in demineralized water for 1 day.

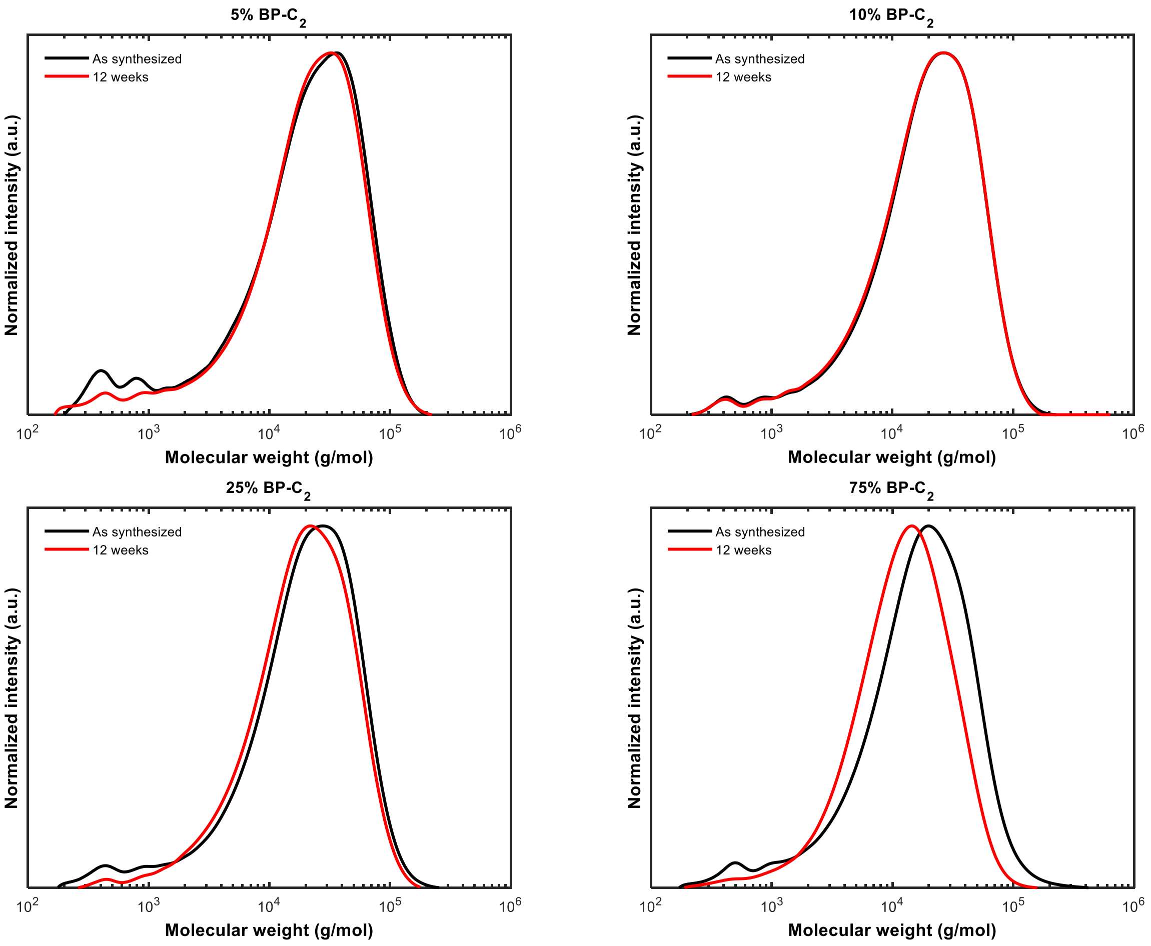

Figure S3. Overview of GPC traces directly after synthesis and after 12 weeks immersion in demineralized water for the polymers **5% BP-C$_2$**, **10% BP-C$_2$**, **25% BP-C$_2$**, and **75% BP-C$_2$**.

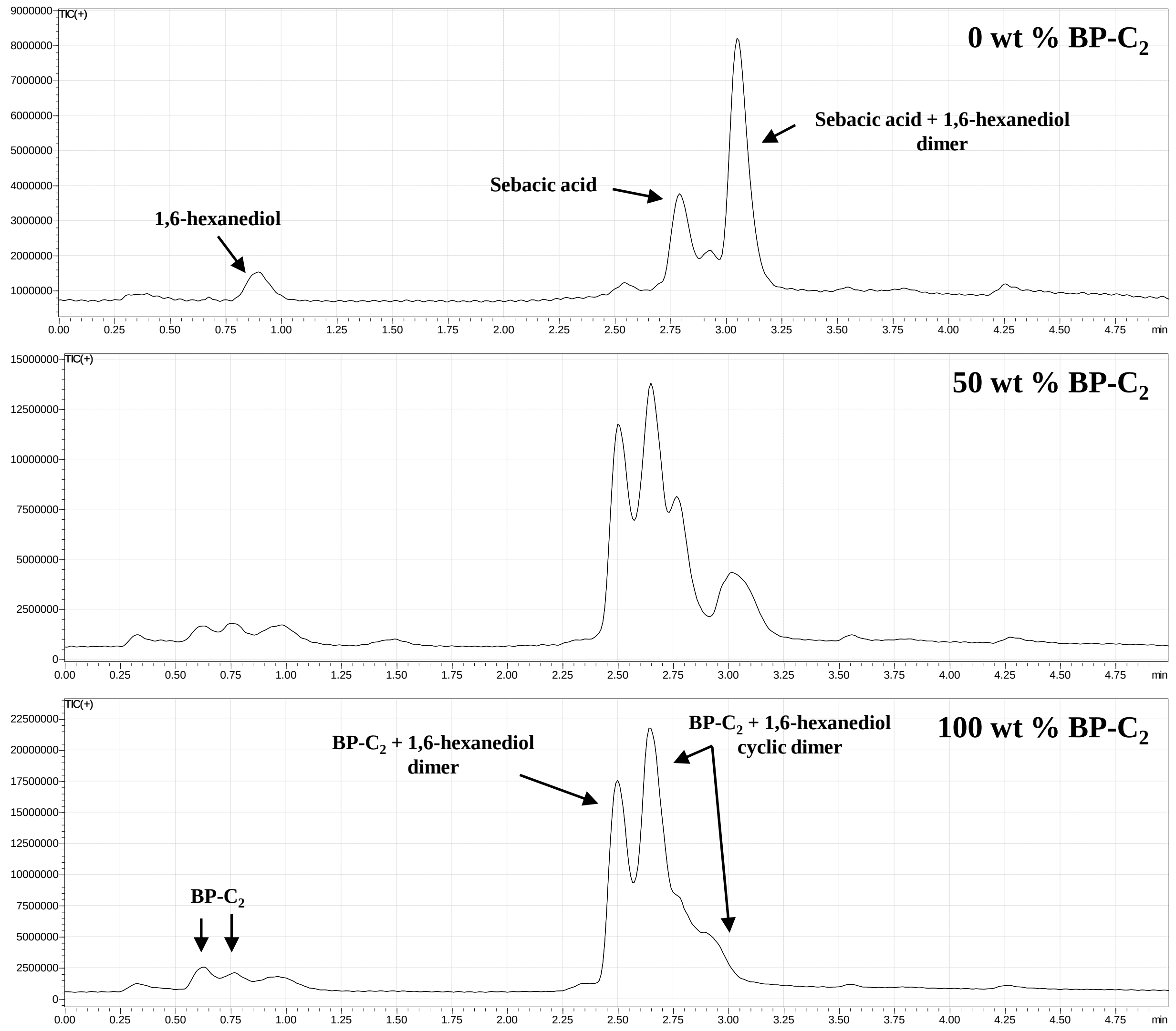

Figure S4. Overview of LC elugrams of the water phase where polymers **0% BP-C_2**, **50% BP-C_2**, **100% BP-C_2** were immersed in for 12 weeks. The arrows denote the suspected species, based on the weight of the fractions detected in MS.

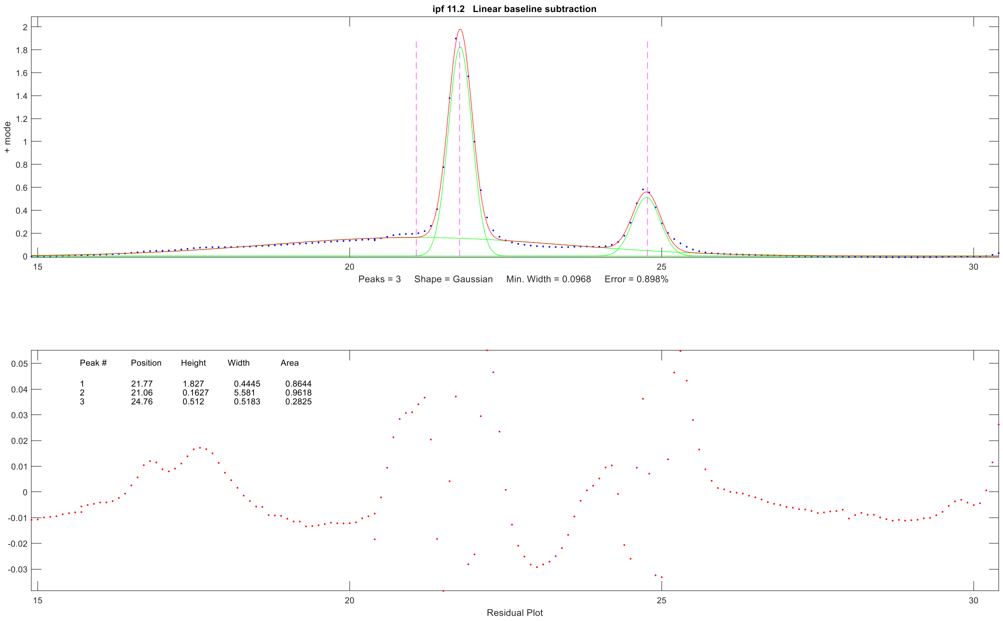

Figure S5. Example of the results of the peak fitting procedure described in the manuscript. Example shown is for polymer **0% BP-C_2.**
